# Comparative Evaluation of Electronic Syringe and Pan Coating Techniques for Loading of FDM 3D Printed Tablets

**DOI:** 10.3390/ph19030411

**Published:** 2026-03-02

**Authors:** Yusra Ahmed, Krisztián Kovács, Krisztina Ludasi, Orsolya Jójárt-Laczkovich, Tamás Sovány

**Affiliations:** 1 Institute of Pharmaceutical Technology and Regulatory Affairs, University of Szeged, Eötvös u 6, H-6720 Szeged, Hungary; 2 Department of Pharmaceutics, Sudan University of Science and Technology, Khartoum P.O. Box 407, Sudan

**Keywords:** fused deposition modeling, polylactic acid, paracetamol, electronic syringe, pan coating, post-printing loading

## Abstract

**Background/Objectives**: 3D printing, particularly fused deposition modeling (FDM), is an emerging technology in pharmaceutical manufacturing, enabling the customization of dose or release rate to individual patient needs. However, finding the appropriate loading method to ensure the stability of the drug and achieve the targeted dose may be challenging. Furthermore, the drug utilization of most loading methods is poor, which results in considerable waste production and increased environmental burden. This study aimed to compare two post-printing drug-loading techniques: electronic syringe deposition and pan coating on FDM-printed polylactic acid (PLA) tablets. PLA is a biodegradable and biocompatible polymer that is widely used in this field due to its mechanical strength and regulatory approval. **Methods**: Tablets with honeycomb-shaped infill (30% and 60% infill densities) were fabricated using PLA filaments, followed by loading with a 15% paracetamol solution via either electronic syringe deposition or pan coating. The resulting tablets were assessed for drug content, weight variation, friability%, surface morphology (SEM), drug distribution (Raman mapping), solid-state characteristics (DSC and FTIR), and dissolution performance. **Results**: The results indicated that pan coating and electronic syringe deposition offered drug utilization up to 88% and 91.7%, respectively, which is superior to conventional soaking methods. Nevertheless, there is a significant difference in drug loading and release rate: pan coating yielded up to 10.14% drug loads and fast release (over 80% in 30 min), while electronic syringe deposition showed lower drug loading up to 4.8% and slower release (less than 80% within 60 min), which could be associated with better mechanical film integrity and higher precision. Both methods met USP standards with a weight loss of less than 1% and maintained the drug’s crystalline state and compatibility with PLA. **Conclusions**: FDM combined with controlled post-printing drug loading presents a rapid, cost-effective, and flexible novel approach for manufacturing personalized immediate-release tablets, with pan coating potentially being more suitable for commercial scalability and electronic syringe offering precise dosing for personalized therapies.

## 1. Introduction

In the pharmaceutical industry, additive manufacturing, like 3D printing technology, is a novel and developing process. The FDA’s approval of Spritam^®^ (levetiracetam) in 2015 and the Investigational New Drug (IND) clearance of Triastek’s T19 in 2021 demonstrate the major advancements of pharmaceutical 3D printing research in the twenty-first century [1].

Customization at a lower cost is one of the key benefits of 3D printing for the pharmaceutical industry. This technology enables the production of medication in unique forms and dosages tailored to each individual, particularly for geriatric and pediatric patients, thereby enhancing patient compliance [2,3]. Moreover, it enables the geometry of the medicine to become more sophisticated, as it provides the user control over the object’s structure by using computer-aided design (CAD). The 3D design is converted into a file format that the printer can recognize (usually STL), and a slicing software slices the object into multiple layers, which will be printed out layer by layer following the specific printing method [4,5]. The precise texture may enable precise control of drug release, thereby opening up novel drug delivery alternatives [6,7].

3D printing techniques include stereolithography (SLA), selective laser sintering (SLS), pressure-assisted microsyringes (PAMs), and fused deposition modeling (FDM). The most common 3D printing process is FDM, which produces 3D objects by extruding a thermoplastic polymeric filament through a heated nozzle, where the filament melts and then deposits layer by layer on the platform to manufacture the product [8,9].

To make drug-loaded devices with FDM, the drug should be included either in the filament, which can be achieved through hot melt extrusion (HME) or impregnation, or loaded into the device post-printing. The preparation of drug-loaded filaments by HME is based on thermal melting of drug/polymer blends, which enables high drug load (up to 80%) [10] but exerts dual thermal stress of the active pharmaceutical ingredients (APIs) during extrusion and printing. In case of impregnation, the drug is introduced into the filament through passive diffusion upon soaking it in a concentrated drug solution. Impregnation is a simple process that requires no additional equipment. However, it does have several drawbacks. It is time consuming, requires highly concentrated drug solutions, results in low drug utilization, there is a risk of drug recrystallization and/or degradation, and the thermal stress during the printing process must still be considered [11,12,13]. To overcome this disadvantage, post-printing impregnation of the device may be a good alternative for loading drugs into 3D printed devices, but additional drawbacks, such as the need for a highly concentrated drug solution, low drug utilization, and hardly controllable final drug content, still remain.

The use of an electronic syringe may be a precise and efficient alternative method that enables accurate deposition of APIs into the printed devices. By regulating parameters such as dispensing volume and speed, the electronic syringe allows for the controlled dispensing of a drug solution. This technique is beneficial for personalized medicine, as it supports dose customization and the incorporation of multiple drugs within a single tablet, but the automation of electronic syringe-based loading is needed to enable the scalability of the manufacturing process [14].

Pan coating may also be considered as a novel loading technique of 3D printed devices based on the deposition of liquid droplets onto a substrate by spraying. It is a high-speed, fully automated process that provides precise control, resulting in reduced material losses. Thus, the goal of this project was the comparison of the effectiveness of pan coating and electronic syringe-based post-printing loading techniques of FDM-printed tablets.

To fulfill this aim, plain printlets were prepared from polylactic acid (PLA). PLA, one of the most popular polymers for 3D printing [15], is made from lactic acid derived from corn and carbohydrates through direct polycondensation of lactic acid, ring-opening polymerization of lactide, and azeotropic dehydrative condensation [16,17]. PLA is an odorless pharmaceutical-grade polymer, which has high mechanical strength [18,19,20] and is biocompatible, biodegradable, shows no toxicity or carcinogenic effects on the human body, and is not metabolized into toxic products [21]. Paracetamol was selected as the model drug, as it is cheap, easily available, and has multiple polymorphic forms [22], which makes it an ideal candidate to study recrystallization-associated risks of solvent-based post-printing loading techniques.

## 2. Results and Discussion

### 2.1. 3D Printing of the Plain PLA Tablets

**Figure 1:**
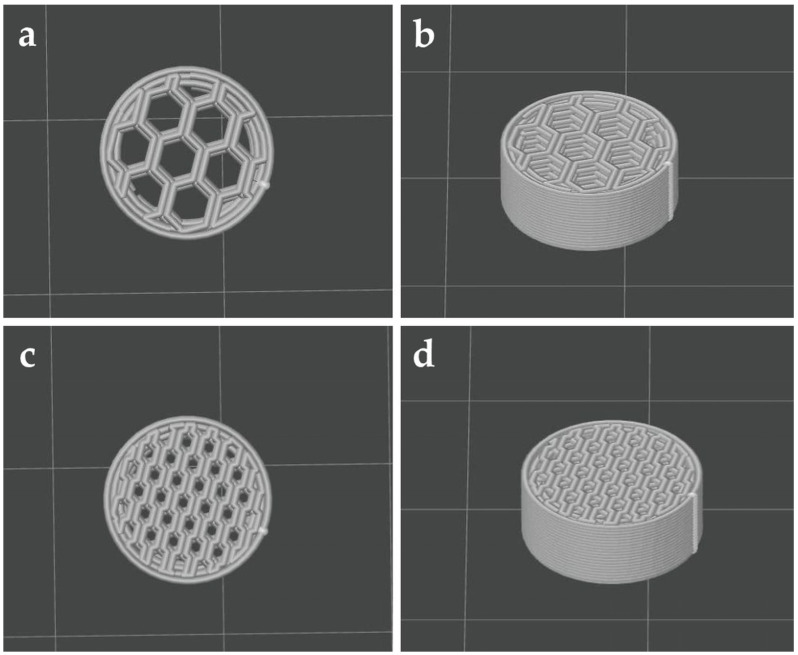
3D representation of the designed tablets: (**a**,**b**) 30% honeycomb-shaped infill; (**c**,**d**) 60% honeycomb-shaped infill.

FDM is the most widely used among 3D printing techniques that have been reported for the preparation of oral solid dosage forms, with more than 70% of studies using this technology [23]. FDM is a fast, effective, and easy-to-use technology that enables the fabrication of tablets with complex geometries. The design of the plain PLA tablet produced by FDM can be seen in Figure 1. The tablets were designed with a honeycomb-shaped internal structure with two different infills (30% and 60%). The main hypothesis was that the honeycomb-shaped internal structure allows for the maximization of the specific surface for drug adsorption, which in the case of 30% infill is associated with big hollow spaces enabling fast penetration of the dissolution medium. In contrast, the narrow pores of devices with 60% infill enables water entrapment by capillary forces, which may help drug accumulation in the internal structure and slow down the drug’s release from the printlet.

### 2.2. Evaluation of Printing Reproducibility

The weight, diameter, and height of plain printlets (n = 20) were measured to evaluate the printing reproducibility (Table 1). FDM demonstrated low variability (RSD < 1%), which suggests excellent printing reproducibility, and the dimensions of the plain printlets are in accordance with the design in the CAD file.

### 2.3. Deposition of Paracetamol Solution

Although it is simple and does not require heating, the soaking method has several drawbacks. Its drug loading efficiency is low, with a significant amount of wasted drug remaining in the solution [9]. Additionally, scaling up this method for industrial use may also be challenging. Therefore, the main objective of this study was to the test the effectiveness of two alternative loading methods in terms of drug loading and utilization efficiency.

An electronic syringe system and pan coating were employed to deposit the paracetamol solution onto the surface of printlets. According to the hypothesis, an electronic syringe offers higher precision of loading by direct injection of the loading solution into pores and therefore minimizes wastage [24,25,26], while pan coating may offer better scalability. The syringe was programmed to dispense controlled volumes of the drug solution directly into designated cavities of the printed structures. Nevertheless, the effective amount of the drug solution was only 100 μL deposited by dropping 20 μL droplets in five cycles. Higher volume may result in spilling out of the solution from the tablet during the loading process, especially in the case of low-infill tablets, leading to low drug loading%. This may cause problems in the commercial manufacturing of high-dose formulations, as increasing the dose may require multiple cycles of loading and drying, which would result in a prolonged processing time and increased risk of nozzle clogging. This may raise the need for other alternative deposition techniques, such as pan coating, that can support higher throughput and better adaptability for commercial-scale production.

Pan coating involves gradual spraying of a precisely measured drug solution onto the surface of printlets rotating in a coating pan. The smaller droplet size and high air volume offers faster drying, enabling the achievement of higher drug loadings, but may result in less uniform drug distribution, which was confirmed by higher surface roughness based on visual inspection. Furthermore, the intensive mechanical impact on printlets during the process resulted in considerable drug loss on abrasion if polymer-free solutions were applied, but the incorporation of HPMC provided strong film-forming properties that enhanced drug adhesion and reduced friability.

### 2.4. Weight Variation and Weight Gain%

Tablets were measured by a calibrated analytical balance at room temperature. All paracetamol-loaded printlets showed acceptable weight variation in agreement with the USP specification (±7.5% in mass range of 130–324 mg) for uncoated tablets, as shown in Table 2 [27,28].

The results confirmed the preliminary hypothesis that faster drying results in higher material deposition during the pan coating process, as pan-coated printlets showed higher weight gains than microsyringe-loaded printlets. The weight gain was 13.07 ± 0.78%, 16.48 ± 0.76%, 5.51 ± 0.21%, and 7.53 ± 0.41% for PC30, PC60, MS30, and MS60, respectively. These findings demonstrate that pan coating may be more effective for applications requiring higher drug loading. The results also confirmed the primary hypothesis that the narrow pores of printlets with 60% infill may keep the liquid with capillary forces and help the entrapment of the drug within the printlets. The observed differences in weight gain percentages also suggest a correlation with drug loading efficiency.

### 2.5. Drug Loading, Content Uniformity, and Drug Utilization

Drug loading and content uniformity were evaluated to assess the consistency and accuracy of the drug incorporation within the dosage forms. These parameters are crucial indicators of the precision of the manufacturing process and formulation quality, directly impacting the therapeutic effectiveness and reproducibility of the product.

**Figure 2:**
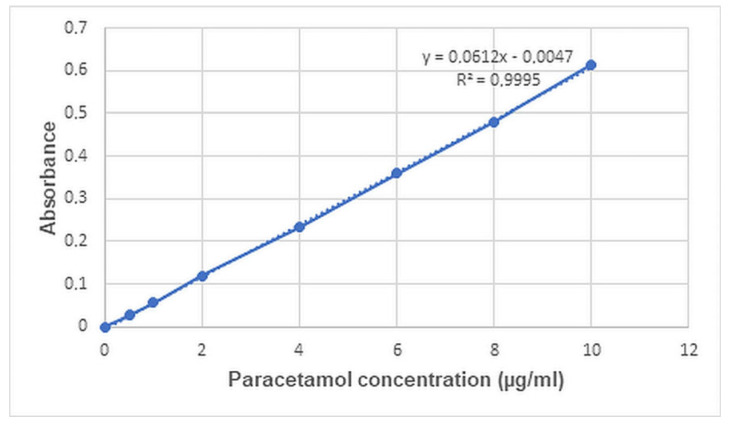
Calibration curve of paracetamol at λmax = 243.

The drug content was evaluated based on UV–Vis spectroscopic measurements using the calibration curve shown in Figure 2.

Interestingly, the results slightly deviated from the results expected based on the weight gain, as PC 30 and MS30 exhibited higher, while PC60 and MS60 exhibited smaller drug content as calculated on the basis of the theoretical composition of the loading solution, which may be due to the different adhesion properties of HPMC and paracetamol. The drug content was 10.11 ± 0.63%, 10.14 ± 0.94%, 4.50 ± 0.15%, and 4.80 ± 0.16% for PC30, PC60, MS30, and MS60, respectively. And the content uniformity was acceptable and fits to the pharmacopeial requirements for all samples. The statistical analysis revealed that the loading method (*x*_1_) exerted significant influence on the drug content (Equation (1)), as drug content decreases by 5.476% if the loading method is changed from pan coating to the microsyringe method.

(1) $$y_{1}=7.387-5.476x_{1}+0.082x_{2}$$

Here, R^2^ = 0.9615, adj R^2^ = 0.9589, and MS Residual = 0.3312.

In contrast, the effect of infill% (*x*_2_) was insignificant, as only a 0.082% increase in content can be expected if the infill% is increased from 0 to +1 level. For detailed statistical results, please see Supplementary Materials.

Besides the achievable drug loading, the effectiveness of drug utilization is also a crucial question, as low drug utilization increases production costs, waste production, and therefore the environmental burden. The soaking method is usually associated with low drug utilization as was confirmed by our comparative experiments, where the theoretical amount in the specific volume of loading solution applied was considered as 100%. The sonication-aided soaking process yields less than 1% drug utilization in the case of both infill%. In contrast, the utilization rate of pan coating was 87.32 ± 4.25% and 88.65 ± 7.93% for PC30 and PC60 printlets, respectively, and even higher in case of the microsyringe loading: 89.19 ± 2.97% and 91.70 ± 2.26% for MS30 and MS 60 printlets, respectively. The difference in the average utilization is smaller than the deviation of the results, which made proper statistical analysis impossible, the corresponding equation (Equation (2)) showed poor fit, and the effect of both factors was insignificant.

(2) $$y_{2}=89.489+1.507x_{1}+0.674x_{2}$$

Here, R^2^ = 0.1070, adj R^2^ = 0.0455, and MS Residual = 25.068.

Nevertheless, the slightly higher yield and lower standard deviations confirm the higher precision of the microsyringe method, while the higher variability of the pan-coated printlets may be attributed to the larger scale of the coating process, where factors such as spray distribution and tablet movement can introduce variability in drug layering compared to the more controlled, small-scale microsyringe method. The results confirm the superiority of both methods over soaking from the aspect of drug utilization and even drug loading, but they still not reach the effectiveness of HME (~80%) [10] from the latter aspect.

### 2.6. Friability

Friability was determined to estimate the mechanical resistance of printlets to abrasion, capping, and chipping during manufacturing, packaging, and transportation. Friability tests indicated no weight loss for PC30 and PC60, while for MS30 and MS60, the friability% was 0.07% ± 0.018 and 0.10% ± 0.008, respectively. This can be attributed to the higher layer thickness of pan-coated samples as well as the more intense mechanical stress on tablets, which can remove the loosely bound particles already during the coating process. In contrast, the use of the microsyringe technique resulted in the formation of a thinner film layer, expected to be friable especially at the edges. Nevertheless, both loading techniques met USP standards with a weight loss of less than 1%.

### 2.7. Scanning Electron Microscopy (SEM)

**Figure 3:**
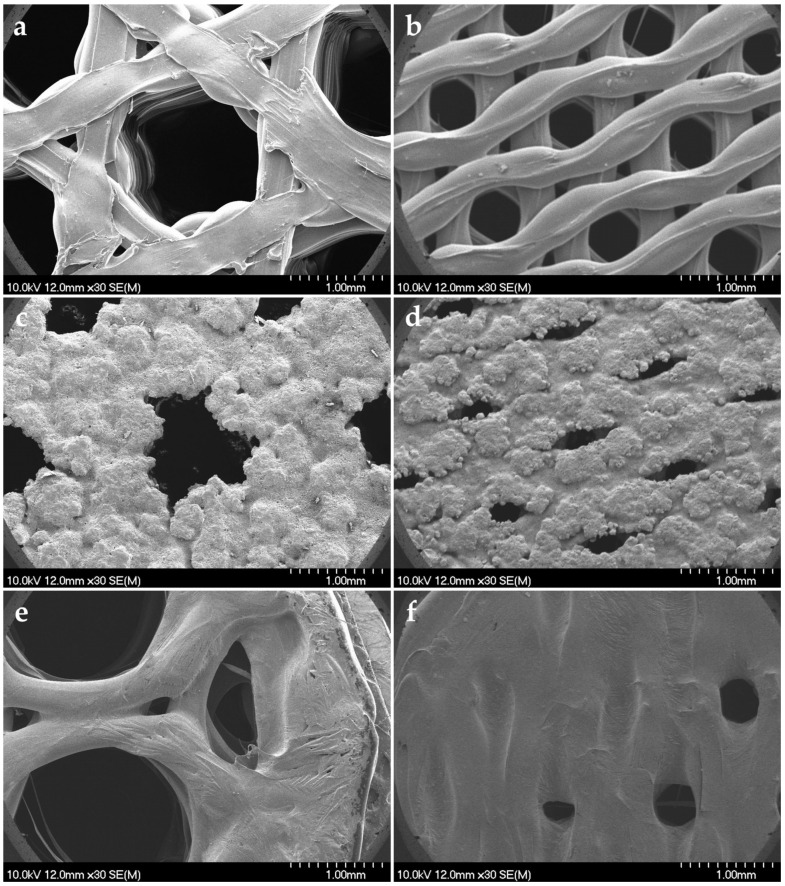
SEM images for plain and loaded PLA tablets: (**a**) plain 30% honeycomb, (**b**) plain 60% honeycomb, (**c**) PC30, (**d**) PC60, (**e**) MS30, and (**f**) MS60.

Scanning electron microscopy (SEM) was performed to observe the surface morphology of the samples before and after loading. The SEM image of the plain printlets is displayed in Figure 3a,b. It shows a generally smooth and uniform surface texture with a few minor printing defects, which were undetectable with normal visual inspection. In contrast, the SEM images show a distinct transformation in surface morphology after loading (Figure 3c–f).

The pan coating process resulted in a considerably rough surface texture with paracetamol crystals partially embedded into a non-uniform HPMC film layer, which could be due to the high atomizing pressure, where the small droplet size and the high volatility of the solvent resulted in fast drying of the loading solution (Figure 3c,d). The fast recrystallization resulted in a partial clogging of the holes for both PC30 and PC60 samples, but despite this, the small droplets may still reach the deeper layer of the printlets during the loading process. In contrast, the bigger droplet size and the relatively slower drying resulted a more uniform distribution of HPMC film with stronger embedding of paracetamol crystals for microsyringe-loaded samples (Figure 3e,f). In the case of MS30 (Figure 3e), the big holes enable the solution to flow down and cover their walls but also increase the risk of material loss, as the solution may flow out on the other side, as was observed during the loading process. MS60 showed a more uniform film layer, with holes being clogged by the HPMC film (Figure 3f), as the smaller pore diameter slows down the penetration of paracetamol solution, so it remains on the surface for a longer time and forms a uniform film layer. The increase in surface roughness and the appearance of additional morphological features suggest the formation of a new layer or the incorporation of active material. Nevertheless, the complete clogging of holes inhibits the penetration of further doses of solution, which may strongly limit the achievable maximal drug load with this process, but this risk can probably be decreased by using less viscous loading solution. For additional micrographs in higher magnification, please see Supplementary Materials.

### 2.8. Qualitative Determination of Drug Content

**Figure 4:**
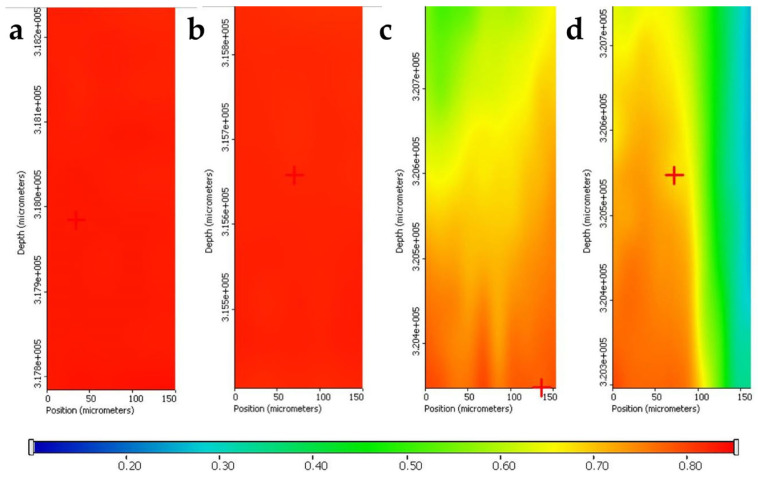
Results of Raman mapping: (**a**) PC30, (**b**) PC60, (**c**) MS30, and (**d**) MS60.

Chemical maps were made to reveal distribution patterns of the drug within the samples. Red areas correspond to paracetamol-rich regions, with green areas indicating lower paracetamol presence and blue regions representing paracetamol-free zones. As can be seen, the fast drying and recrystallization resulted in clear paracetamol enrichment in the top 50 µm of the pan-coated printlets (Figure 4a,b), while the drug can be found in less concentrated form in the case of the microsyringe-loaded samples (Figure 4c,d), probably due to the slower evaporation of the solvent, which enabled better embedding of the drug into the polymer matrix.

### 2.9. Differential Scanning Calorimetry (DSC)

**Figure 5:**
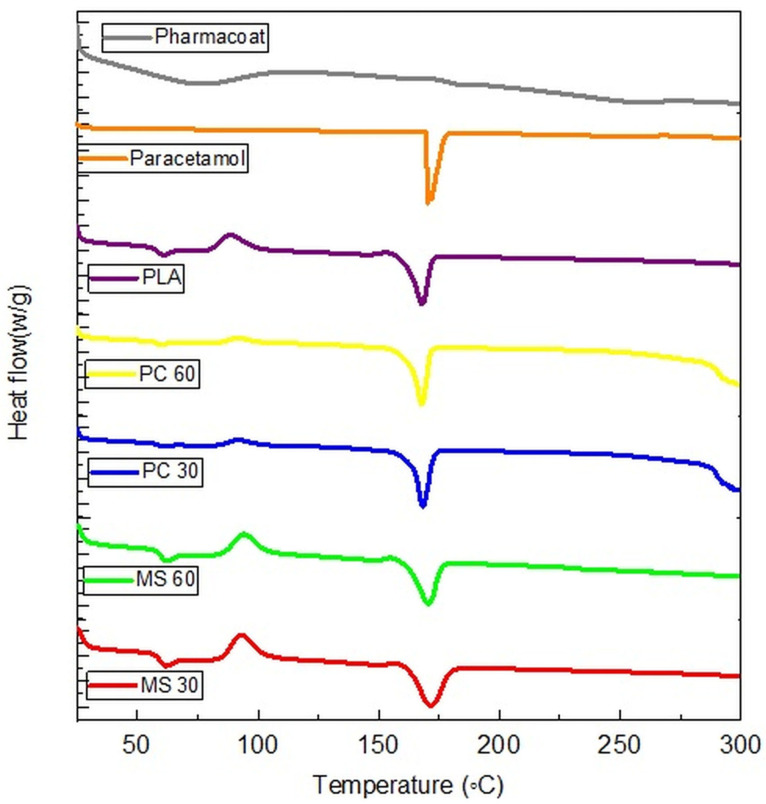
DSC thermograms of plain and loaded PLA tablets.

A secondary objective of the present work is if recrystallization due to solvent evaporation may influence the polymorphic form of the drug and thus influence the stability of the dosage form. Crystalline paracetamol powder showed a sharp endothermic melting peak with an onset of 169.0 °C and a peak of 171–172 °C [29]. PLA filaments were characterized by multiphase transitions, involving a glass transition with an endothermic peak (∼66 °C), a cold crystallization (∼110–120 °C), and a final melting (∼170.5 °C), in agreement with previous reports on pure PLA systems [16,30]. The absence of a sharp melting peak for HPMC confirms its amorphous nature [31]. The paracetamol has completely recrystallized from the loading solutions and can be found in crystalline form within all loaded tablets, as shown in Figure 5. As can be seen, the melting point of paracetamol and PLA is very close to each other, and the melting peaks cannot be distinguished clearly in the loaded samples. Nevertheless, a slight left shift of the paracetamol peak may be presumed in the case of PC30 and PC60, which may be associated with the smaller particle size of the drug.

### 2.10. Structural Analysis by Fourier Transform Infrared Spectroscopy (FT-IR)

**Figure 6:**
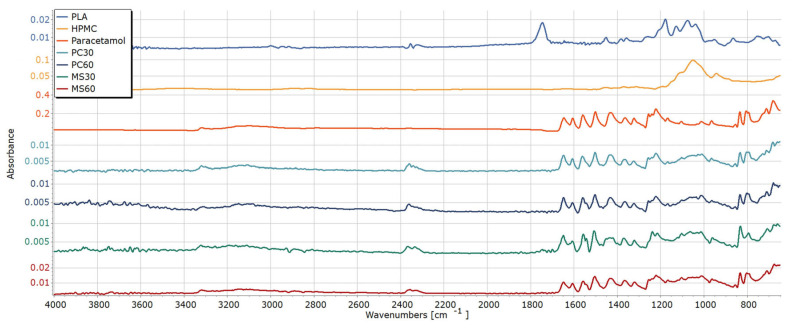
FTIR spectra of plain and loaded PLA tablets.

The FTIR spectrum of paracetamol exhibits characteristic absorption bands corresponding to its functional groups (Figure 6). A band is observed around 3300–3350 cm^−1^, attributed to O–H stretching of the phenolic group and N–H stretching of the amide group. A strong absorption band at approximately 1650 cm^−1^ is assigned to C=O stretching of the amide group. The aromatic C=C stretching vibrations occur around 1500–1600 cm^−1^. Additionally, bands at 1250–1320 cm^−1^ correspond to C–N stretching, and those near 1150–1220 cm^−1^ are due to C–O stretching of the phenol. Characteristic out-of-plane aromatic C–H bending is also observed in the 700–900 cm^−1^ region. The unprocessed PLA filament depicted characteristic bands at around 1745 cm^−1^ (C=O stretching), 1080–1180 cm^−1^ (C–O–C bending), 1360 cm^−1^ (C–H bending), and 1450 cm^−1^ (CH_3_ bending) [32,33,34,35]. In addition, bands at 860 cm^−1^ and 755 cm^−1^ represent the crystalline and amorphous fractions of PLA, respectively [36]. Nevertheless, the characteristic peaks of PLA cannot be identified in the loaded samples, possibly due to the low penetration ability (approx. 10 µm) of infrared waves into the samples [37], which also supports that the PLA surface is completely covered by paracetamol crystals embedded in HPMC film. The characteristic peaks of paracetamol were identical in all tested samples, suggesting that no polymorphic changes or considerable chemical interactions occurred during the loading process and confirming the compatibility of PLA with paracetamol [38].

### 2.11. Drug Release Studies

**Figure 7:**
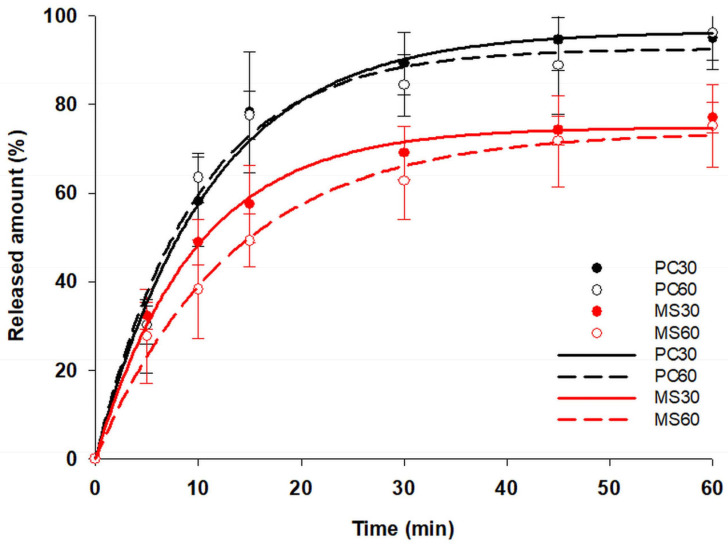
Drug release profile of printed tablets.

Dissolution data from the paracetamol printlets are shown in Figure 7. The dissolution followed first-order kinetics for all samples (R^2^ equals 0.9876, 0.9829, 0.9960, and 0.9914 for PC30, PC60, MS30, and MS60, respectively), and pan-coated samples fit to the requirements of immediate-release dosage forms as per the USP monograph [27], since more than 80% of the drug was released in 30 min. The difference and similarity factors f1 and f2 were 5 and 70.39, respectively, so the dissolution curves can be considered as equivalent. The fast release may be due to the absence of a uniform HPMC film layer that would modulate the drug release, resulting in a relatively rapid onset of action [39]. In contrast, for MS30 and MS60, less than 80% of the drug was released within an hour. And the calculated f1 factor was 21.14 and 26.28, while the f2 factor was 40.61 and 34.69 compared to the pan-coated samples for samples MS30 and MS60, respectively, which confirms the difference in the dissolution kinetics. Compared to each other, the f1 and f2 factors were 9.8 and 60.48, respectively, which shows the similarity of the release from the microsyringe-loaded samples. The decreased release rate may be due to the way that paracetamol was incorporated into the printlets, the stronger embedding of paracetamol into the more uniform HPMC film, and the possible increased penetration of the drug solution into its pores. The results suggests that the release rate can be tailored well by the setting of infill%, HPMC concentration, and film uniformity.

## 3. Materials and Methods

### 3.1. Materials

PLA filaments were purchased from Formlabs (Somerville, MA, USA), the paracetamol used as the model drug was kindly gifted by Gedeon Richter Plc. (Budapest, Hungary), and the hydroxypropyl-methyl-cellulose (HPMC, Pharmacoat 606) used as a film-forming agent to stabilize paracetamol on the printlet surface was purchased from Shin-Etsu (Tokyo, Japan). Other materials used were reagent grade.

### 3.2. Methods

#### 3.2.1. Geometry Design and Printing

The tablet geometry was designed using CAD software (Autodesk Fusion 360, Mill Valley, CA, USA). Briefly, a round object (diameter: 10.0 mm; height: 4.5 mm) was designed, with various infill patterns and densities. The design was then exported as an STL file into the slicing software (ORCA slicer, Shenzhen, China). A Bambulab A1 (Bambu Lab Co., Ltd., Shenzhen, China) FDM printer was used to fabricate the plain PLA tablets, with the following printing parameters: extrusion temperature (210 °C), build plate temperature (60 °C), infill percentage (30% and 60%), infill pattern (honeycomb), nozzle diameter (0.4), layer height (0.2), travel speed (2000 mm/s), and printing speed (500 mm/s), with the aid of a raft. Infill patterns and percentages tested were selected based on preliminary experiments, where the surface area of various patterns was calculated and the structure of printlets with an infill% from 10 to 90% was studied. It was revealed that for very low infills of 10 or 20%, the large hole size and fewer internal contact points resulted in insufficient drug adsorption, while in the case of large infills over 60%, the infill pattern started to distort, as lines are too close, over-extrusion becomes obvious, and, subsequently, pressure inside the nozzle increases (limitation of the printer itself).

#### 3.2.2. Evaluation of Printing Reproducibility

In total, 20 plain tablets were printed, and their weight, diameter, and height were measured with the aid of a tablet tester (Sotax MT50, Aesch, Switzerland) to investigate the printing reproducibility. Results were expressed as the mean, standard deviation (SD), and relative standard deviation (RSD).

#### 3.2.3. Preparation of Loading Solution

The loading solution was prepared by dissolving 15 g of paracetamol in 100 mL of 96% ethanol, followed by the dissolution of 5 g of HPMC in the same solution. The role of HPMC is the stabilization of the API on the printlets, since in preliminary experiments, intensive abrasion of the loaded drug was observed, especially in pan coating experiments. The solution was stirred at 500 rpm for 60 min in a Multi-HS 6 Digital magnetic stirrer (VELP Scientifica Srl, Usmate Velate, Italy) until both components dissolved completely to ensure homogeneity. The 15% *w*/*v* paracetamol was selected considering the 190.6 g/L solubility of the drug in 96% ethanol [40], high enough to enable high drug loading but below the saturation limit, preventing accidental recrystallization and nozzle clogging during the loading procedure.

#### 3.2.4. Loading of Paracetamol Solution by Simple Soaking

The plain PLA printlets were immersed into the ethanolic loading solution, and the beaker was covered with parafilm to avoid the evaporation of the ethanol and kept in the water bath of an Almasonic P ultrasonic bath (Elma Schmidbauer GmbH, Singen, Germany) at a temperature of 50 °C for 8 h. The drug-loaded tablets were then placed on a tray and dried overnight under ambient conditions (25 °C and 40% rH) for further investigation.

#### 3.2.5. Deposition of Paracetamol Solution by Electronic Syringe

The ethanolic loading solution was deposited on top of the cavities of the plain PLA printlets via the automatic syringe system of a Dataphysics OCA 20 device (Dataphysics, Fielderstadt, Germany). The solution was dispensed from a 500 µL Hamilton syringe connected to a needle tip of 1.54 mm in diameter with a dosing rate of 20 µL/s. The needle and dosing rate were selected based on the viscosity of the loading solution to enable accurate drop formation and fast dosing with a dropping interval, enabling user intervention if needed. Two types of paracetamol tablets were produced by loading printlets of 30% and 60% honeycomb-shaped infills, referred to as MS 30 and MS 60, respectively.

#### 3.2.6. Loading of Paracetamol Solution by Pan Coating Technique

The pan coating process was conducted in a 4M8 Pancoat perforated drum coater (ProCepT, Zelzate, Belgium). The plain PLA printlets with different infills were mixed with microcrystalline cellulose-based placebo tablets of the same dimensions as the printlets to ensure the required load of the pan coater. The ethanolic loading solution was dispensed on the surface of tablets preheated in the pan at 50 °C for 10 min to eliminate surface moisture and ensure the required core temperature. Then, 275 mL of coating solution was sprayed at a rate of 25 mL/min with an atomizing pressure of 2.0 bar, while heated air at 45 °C facilitated solvent evaporation, and the air speed was 0.7 m/s. The speed of the coating pan was set to 10 rpm. After the coating was completed, the tablets were dried for 15 min at 50 °C to remove residual moisture. The overall process including preheating, coating, and drying lasted for 36 min. The samples were then cooled under ambient conditions (25 °C and 40% rH) for 24 h before being evaluated. Two types of paracetamol tablets were produced, PC 30 and PC 60, for printlets with 30% and 60% honeycomb-shaped infills, respectively.

#### 3.2.7. Weight Variation and Weight Gain%

Ten printlets were weighed individually on an analytical scale (Sartorius AG, Göttingen, Germany) before and after loading. The average weight and percentage of weight gain were calculated.

#### 3.2.8. Drug Utilization, Drug Loading, and Content Uniformity

Eight printlets were weighed individually, and each of them was placed in 250 mL of pH 5.8 phosphate buffer solution. The samples were kept in a magnetic stirrer with a stirring speed of 400 rpm for 120 min to ensure the complete extraction of paracetamol. The obtained solutions were diluted to the required concentration, and the absorbance was measured at 243 nm with a GENESYS 10S UV–Vis spectrometer (ThermoFisher Scientific, Waltham, MA, USA). The drug content and content uniformity were calculated using the standard calibration curve of paracetamol, and drug loading is expressed as the content % of the printlet weight, while drug utilization was expressed as the content % of the original drug content of the applied volumes of the loading solution.

#### 3.2.9. Friability

The friability test was carried out because both loading techniques could lead to the accumulation of the drug on the printlet surface, which may result in the loss of paracetamol content during handling/packaging. The loaded printlets were weighed and placed into a TA100 friabilator (Erweka, Langen, Germany). The tester rotated for 4 min at 25 rpm. The tablets were weighed again, and the relative weight loss was calculated.

#### 3.2.10. Scanning Electron Microscopy (SEM)

The morphology of printlets was assessed using scanning electron microscopy (SEM) to analyze the printing quality of plain PLA printlets and to reveal changes in the tablet surface after impregnation. The morphology was analyzed using an S4700 SEM apparatus (Hitachi Scientific Ltd., Tokyo, Japan). The samples were fixed with a conductive carbon double adhesive tape, and a conductive gold coating layer was applied to further increase the surface conductivity of samples, using a Quorum Q150R S plus sputter coater (Quorum Technologies Ltd., Laughton, UK) under high-vacuum conditions in an argon environment. The samples were analyzed with an accelerating voltage of 10 kV, a beam current of 10 µA, and an air pressure range of 1.3 to 13.0 mPa. The SEM images of the samples were captured with 30× magnification.

#### 3.2.11. Qualitative Determination of Drug Content

To visualize drug concentration and location in the samples, Raman mapping was used. Ten measurement points were taken for each sample. An XRD Dispersive Raman microscope (ThermoFisher Scientific Inc., Waltham, MA, USA) was used for the measurement, which included a 780 nm diode laser and a CCD camera. Depth profiling was carried out using a step size of 19 μm and a vertical “immersion” (Z-axis) of 50 μm. The exposure time was set at 6 s, yielding 12 scans per spectrum from 3500 to 200 cm^−1^. The chemical evaluation was performed using OMNIC for Dispersive Raman 8.2 software (ThermoFisher Scientific Inc., Waltham, MA, USA). The individual spectrum of paracetamol was used as a reference while creating the chemical map.

#### 3.2.12. Differential Scanning Calorimetry (DSC)

A Mettler Toledo DSC 821e instrument (Mettler Inc., Schwerzenbach, Switzerland) was used to analyze the thermal stability and solid state of samples. The precise amount of each sample (approximately 3–5 mg) was weighed and placed in a sealed aluminum pan. The samples were heated from 25 to 300 °C at a constant heat flow of 10 °C/min under a flow of 50 mL/min of an inert gas (nitrogen). An empty aluminum pan was used as a reference. OriginPro 8.5 software (OriginLab Corporation, Northampton, MA, USA) was used to visualize the results.

#### 3.2.13. Structural Analysis by Fourier Transform Infrared Spectroscopy (FTIR)

An FTIR spectrometer was utilized to characterize the functional groups of samples before and after impregnation to assess any potential interactions among the components of each sample. The FTIR spectra were recorded using Avatar 330 FT-IR (Thermo Fisher Scientific Inc., Waltham, MA, USA), covering a wavenumber range from 4000 to 400 cm^−1^ with an optical resolution of 4 cm^−1^. The spectra were obtained from 128 scans. To evaluate the results, SpectraGryph software (version 1.2.15., F. Menges Software Entwicklung, Germany) was used.

#### 3.2.14. Drug Release Studies

The dissolution study was carried out using an Erweka DT700 USP II dissolution testing apparatus (Erweka GmbH, Langen, Germany). Here, 900 mL of dissolution medium (5.8 phosphate buffer) was used with a temperature of 37 ± 0.5 °C and 50 rpm as the rotation speed. At intervals of 5, 10, 15, 30, 45, and 60 min, 5 mL of the sample was withdrawn. After appropriate dilution, the samples were analyzed at 243 nm using a GENESYS 10S UV–Vis spectrometer (ThermoFisher Scientific, Waltham, MA, USA). All measurements were performed in triplicate, and the results were expressed as the mean ± standard deviation.

The kinetic model was fitted with Sigmaplot v.12.1 (Systat Software Inc., San Jose, CA, USA), and difference (f1) and similarity (f2) factors were calculated according to Equations (3) and (4), respectively.

(3) $$f_{1}=\left\{\frac{\sum_{t-1}^{n}\left|R_{t}-T_{t}\right|}{\sum_{t-1}^{n}R_{t}}\right\}\times 100$$

(4) $$f_{2}=50\times log\left({\left\lceil 1+\frac{1}{n}\sum_{t=1}^{n}{\left(R_{t}-\left.T_{t}\right)\right.}^{2}\right\rceil }^{-0.5}\times 100\right)$$

Here, R_t_ and T_t_ are the reference and test profiles, respectively. f1 represents the percent difference at each timepoint, and values between 0 and 15 represents similar curves, where 0 means no difference, while f2 values between 50 and 100 also suggests that curves are similar, and 100 represents identical curves.

#### 3.2.15. Statistical Analysis

The results were evaluated according to a 2^2^ full factorial design, where the loading method (*x*_1_) served as the qualitative factor, with pan coating and the microsyringe method as low (−1) and high (+1) levels, respectively. Infill percent (*x*_2_) was considered as a continuous factor, with 30% and 60% infills as low (−1) and high (+1) levels, respectively. Drug loading (*y*_1_) and drug utilization (*y*_2_) were analyzed as output parameters. The analysis was performed using Tibco Statistica v. 13.6 (Tibo Software Inc., Palo Alto, CA, USA).

## 4. Conclusions

This study successfully compared the effectiveness of alternative post-print loading techniques of FDM-printed tablets: electronic syringe deposition and pan coating. The method developed in this work for manufacturing tablets has some advantages over traditional impregnation by soaking and HME. Compared to traditional impregnation, both loading techniques reduced the processing time significantly, minimized drug wastage, and increased the achievable drug loading, but from this aspect, the effectiveness of HME was still not achieved. Nevertheless, compared to HME, drug degradation associated with high temperatures can be avoided. Furthermore, both electronic syringe and pan coating methods proved to have unique advantages; pan coating delivered tablets with faster release and potential scalability, positioning it as a more feasible approach for industrial production, while the syringe method achieved higher drug loading, and the more uniform film structure may enable slower release profiles, enabling the control and tailoring of the release rate.

Importantly, both techniques preserved the crystalline state of paracetamol and maintained its compatibility with PLA, as confirmed by SEM, DSC, and FTIR analyses. The use of PLA as a printable, pharmaceutical-grade material further supports the biocompatibility and practicality of this approach. Overall, the integration of FDM 3D printing with post-processing drug loading, particularly pan coating, presents a cost-effective, flexible, and scalable pathway for the production of personalized oral dosage forms.

## Figures and Tables

**Table 1:** Results of printing reproducibility.

Parameter	Diameter (mm)	Height (mm)	Weight (mg)
30% Honeycomb			
Mean	10.22	4.452	287.04
SD *	0.075	0.026	1.95
RSD **	0.734	0.581	0.68
60% Honeycomb			
Mean	10.14	4.438	277.2
SD	0.060	0.022	2.058
RSD	0.590	0.488	0.74

* SD = Standard deviation ** RSD = Relative Standard Deviation.

**Table 2:** Results for weight variation test.

	PC 30	PC 60	MS 30	MS 60
Mean	323.5 mg	322.9 mg	301.73 mg	296.8 mg
SD *	7.9	7.7	2.8	2.3
RSD **	2.44	2.37	1.02	0.758

* SD = Standard deviation ** RSD = Relative Standard Deviation.

## Data Availability

The raw data supporting the conclusions of this article will be made available by the authors on request.

## Supplementary Materials

The following supporting information can be downloaded at: https://www.mdpi.com/article/10.3390/ph19030411/s1, Table S1: Full ANOVA table of effect estimates on drug utilization if all parameters are considered; Figure S1: Residual plot analysis on drug utilization if all parameters are considered; Table S2: Full ANOVA table of effect estimates on drug utilization if interaction term is eliminated; Figure S2: Residual plot analysis on drug utilization if interaction term is eliminated; Table S3: Full ANOVA table of effect estimates on drug content if all parameters are considered; Figure S3: Residual plot analysis on drug content if all parameters are considered; Table S4: Full ANOVA table of effect estimates on drug content if interaction term is eliminated; Figure S4: Residual plot analysis on drug content if interaction term is eliminated; Table S5: Full ANOVA table of effect estimates on drug content if all insignificant terms are eliminated; Figure S5: Residual plot analysis on drug content if all insignificant terms are eliminated; Figure S6: MS30 at 30× magnification; Figure S7: MS30 at 100× magnification; Figure S8: MS30 at 250× magnification; Figure S9: MS30 at 500× magnification; Figure S10: MS60 at 30× magnification; Figure S11: MS60 at 100× magnification; Figure S12: MS60 at 250× magnification; Figure S13: MS60 at 500× magnification; Figure S14: PC30 at 30× magnification; Figure S15: PC30 at 100× magnification; Figure S16: PC30 at 250× magnification; Figure S17: PC30 at 500× magnification; Figure S18: PC60 at 30× magnification; Figure S19: PC60 at 100× magnification; Figure S20: PC60 at 250× magnification.
