# Supplementary material for: Comparative Evaluation of Electronic Syringe and Pan Coating Techniques for Loading of FDM 3D Printed Tablets
Source: Pharmaceuticals (Basel). 2026 Mar 2;19(3):411. doi: 10.3390/ph19030411 (PMC13028998; doi:10.3390/ph19030411)
Supplement: Supplementary file 1 [file pharmaceuticals-19-00411-s001.zip › pharmaceuticals-4150273-supplementary.pdf]

Table S1: Full ANOVA table of effect estimates on drug utilization if all parameters is considered.

Factor	Effect Estimates; Var.:Drug utilization%; R-sqr=,10953; Adj:,01413 (Spreadsheet2-for DoE in Workbook1) 2**(2-0) design; MS Residual=23,2185 DV: Drug utilization%								
	Effect	Std.Err.	t(28)	p	-95,% Cnf.Limt	+95,% Cnf.Limt	Coeff.	Std.Err. Coeff.	-95,% Cnf.Limt	+95,% Cnf.Limt
Mean/Inter c.	89,20813	0,851809	104,7279	0,000000	87,46327	90,95298	89,20813	0,851809	87,46327	90,95298
(1)Loading method	2,45125	1,703618	1,4388	0,161279	-1,03845	5,94095	1,22562	0,851809	-0,51923	2,97048
(2)Infill%	1,91000	1,703618	1,1211	0,271750	-1,57970	5,39970	0,95500	0,851809	-0,78985	2,69985
1 by 2	0,58250	1,703618	0,3419	0,734965	-2,90720	4,07220	0,29125	0,851809	-1,45360	2,03610

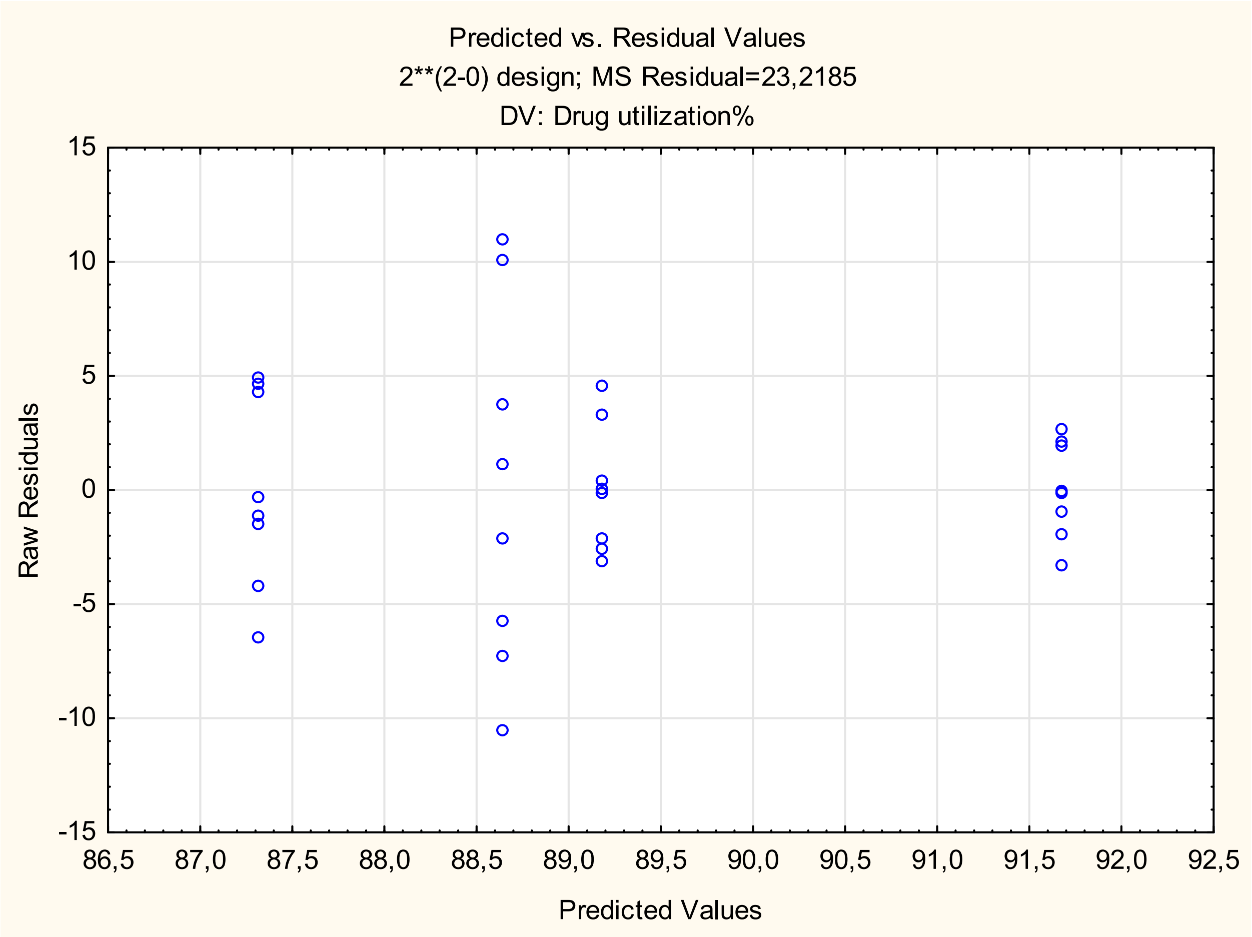

Figure S1: Residual plot analysis on drug utilization if all parameters is considered.

Table S2: Full ANOVA table of effect estimates on drug utilization if interaction term is eliminated.

Factor	Effect Estimates; Var.:Drug utilization%; R-sqr=,10581; Adj:,04415 (Spreadsheet2-for DoE in Workbook1) 2**(2-0) design; MS Residual=22,51146 DV: Drug utilization%									
	Effect	Std.Err.	t(29)	p	-95,% Cnf.Limt	+95,% Cnf.Limt	Coeff.	Std.Err. Coeff.	-95,% Cnf.Limt	+95,% Cnf.Limt
Mean/Inter c.	89,20813	0,838739	106,3598	0,000000	87,49271	90,92354	89,20813	0,838739	87,49271	90,92354
(1)Loading method	2,45125	1,677478	1,4613	0,154693	-0,97958	5,88208	1,22562	0,838739	-0,48979	2,94104
(2)Infill%	1,91000	1,677478	1,1386	0,264183	-1,52083	5,34083	0,95500	0,838739	-0,76041	2,67041

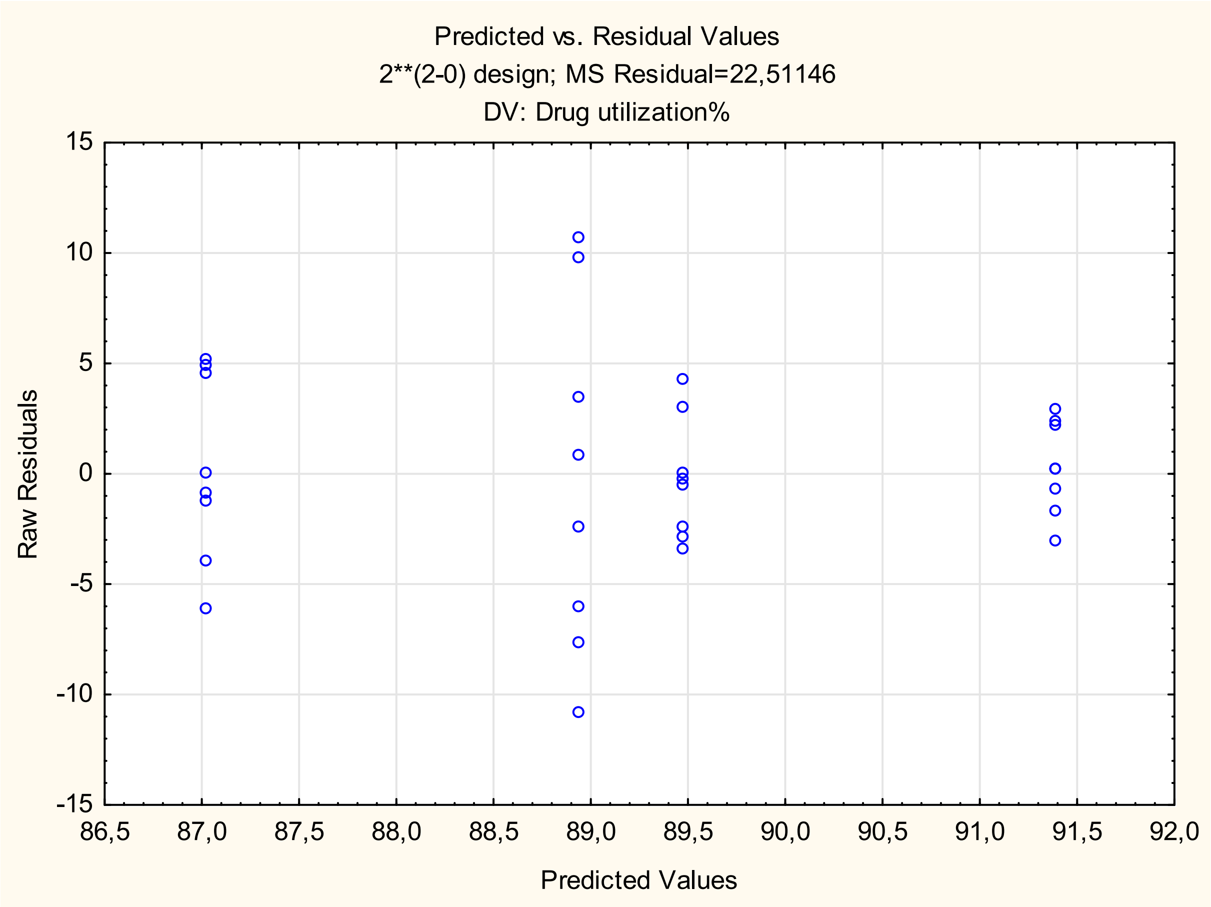

Figure S2: Residual plot analysis on drug utilization if interaction term is eliminated.

Table S3: Full ANOVA table of effect estimates on drug content if all parameters is considered.

Factor	Effect Estimates; Var.:Drug content (%); R-sqr=,9621; Adj:,95804 (Spreadsheet2-for DoE in Workbook1) 2**(2-0) design; MS Residual=,3379285 DV: Drug content (%)									
	Effect	Std.Err.	t(28)	p	-95,% Cnf.Limt	+95,% Cnf.Limt	Coeff.	Std.Err. Coeff.	-95,% Cnf.Limt	+95,% Cnf.Limt
Mean/Inter c.	7,38439	0,102763	71,8583	0,000000	7,17389	7,59489	7,38439	0,102763	7,17389	7,59489
(1)Loading method	-5,47555	0,205526	-26,6416	0,000000	-5,89656	-5,05455	-2,73778	0,102763	-2,94828	-2,52728
(2)Infill%	0,16438	0,205526	0,7998	0,430568	-0,25662	0,58538	0,08219	0,102763	-0,12831	0,29269
1 by 2	0,13329	0,205526	0,6485	0,521938	-0,28772	0,55429	0,06664	0,102763	-0,14386	0,27714

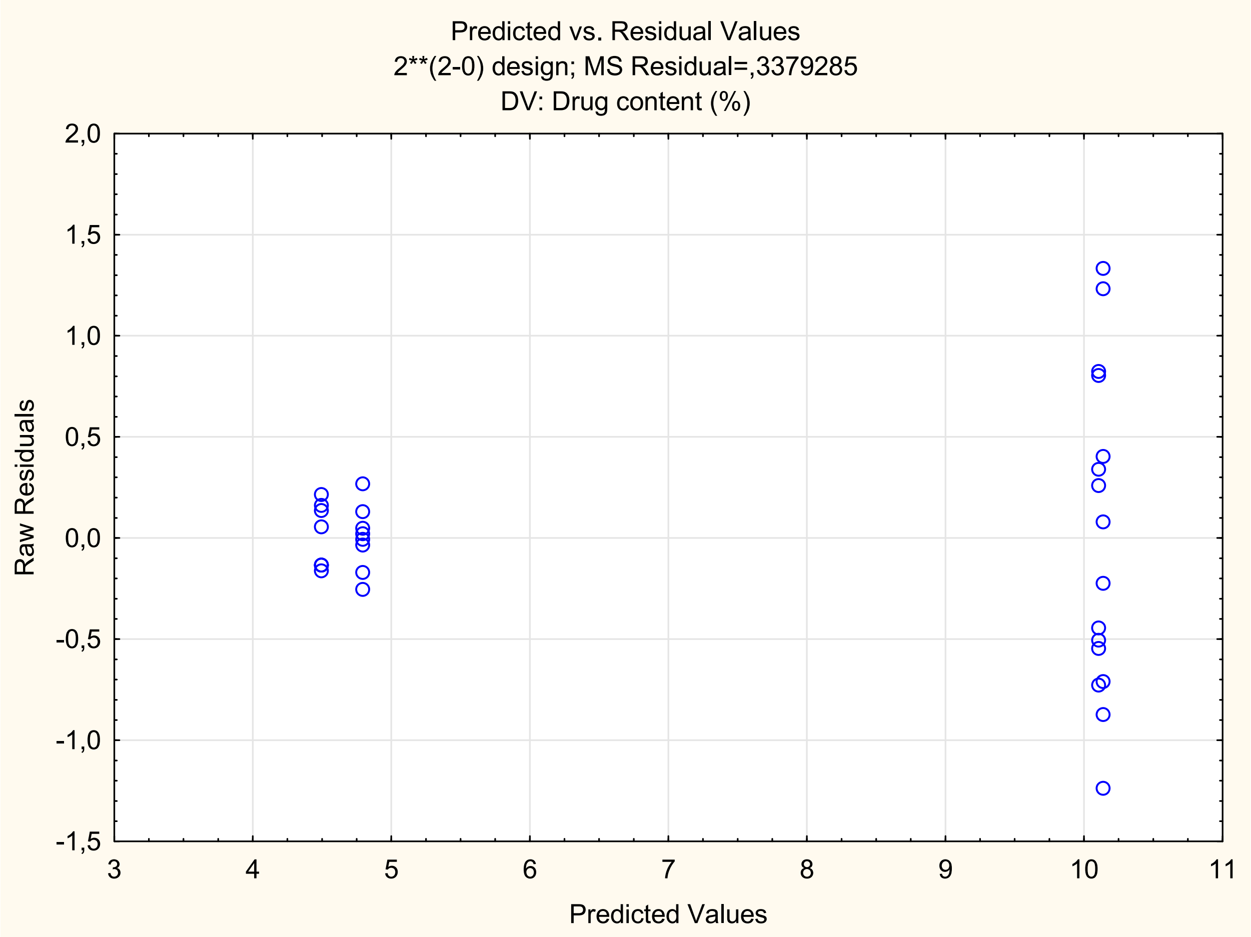

Figure S3: Residual plot analysis on drug content if all parameters is considered.

Table S4: Full ANOVA table of effect estimates on drug content if interaction term is eliminated.

Factor	Effect Estimates; Var.:Drug content (%); R-sqr=,96153; Adj:,95888 (Spreadsheet2-for DoE in Workbook1) 2**(2-0) design; MS Residual=,3311765 DV: Drug content (%)									
	Effect	Std.Err.	t(29)	p	-95,% Cnf.Limt	+95,% Cnf.Limt	Coeff.	Std.Err. Coeff.	-95,% Cnf.Limt	+95,% Cnf.Limt
Mean/Inter c.	7,38439	0,101731	72,5872	0,000000	7,17633	7,59245	7,38439	0,101731	7,17633	7,59245
(1)Loading method	-5,47555	0,203463	-26,9118	0,000000	-5,89168	-5,05943	-2,73778	0,101731	-2,94584	-2,52971
(2)Infill%	0,16438	0,203463	0,8079	0,425727	-0,25175	0,58050	0,08219	0,101731	-0,12588	0,29025

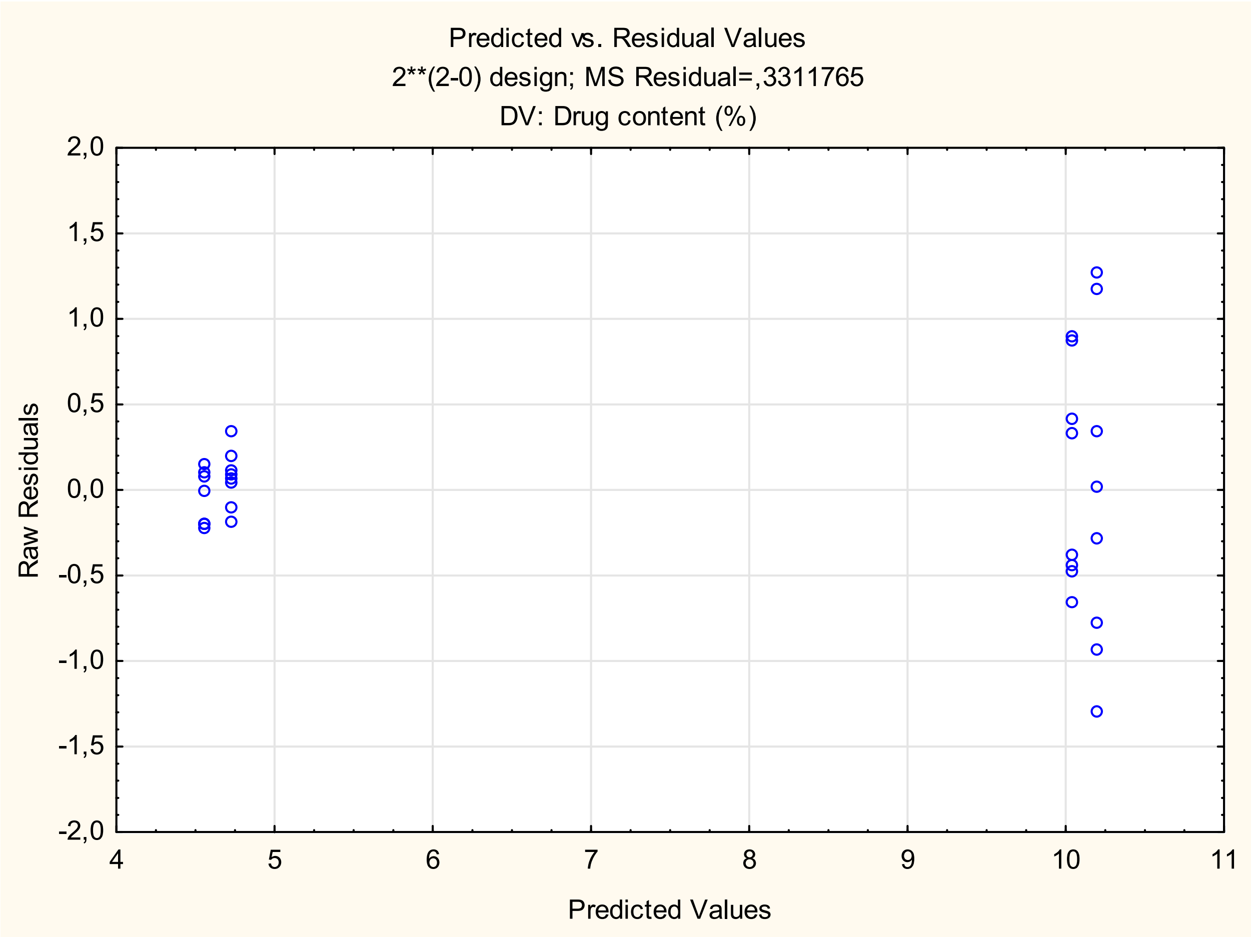

Figure S4: Residual plot analysis on drug content if interaction term is eliminated.

Table S5: Full ANOVA table of effect estimates on drug content if all insignificant term is eliminated.

7,38439	0,101141	73,0110	0,000000	7,17783	7,59095	7,38439	0,101141	7,17783	7,59095
-5,47555	0,202282	-27,0690	0,000000	-5,88867	-5,06244	-2,73778	0,101141	-2,94433	-2,53122

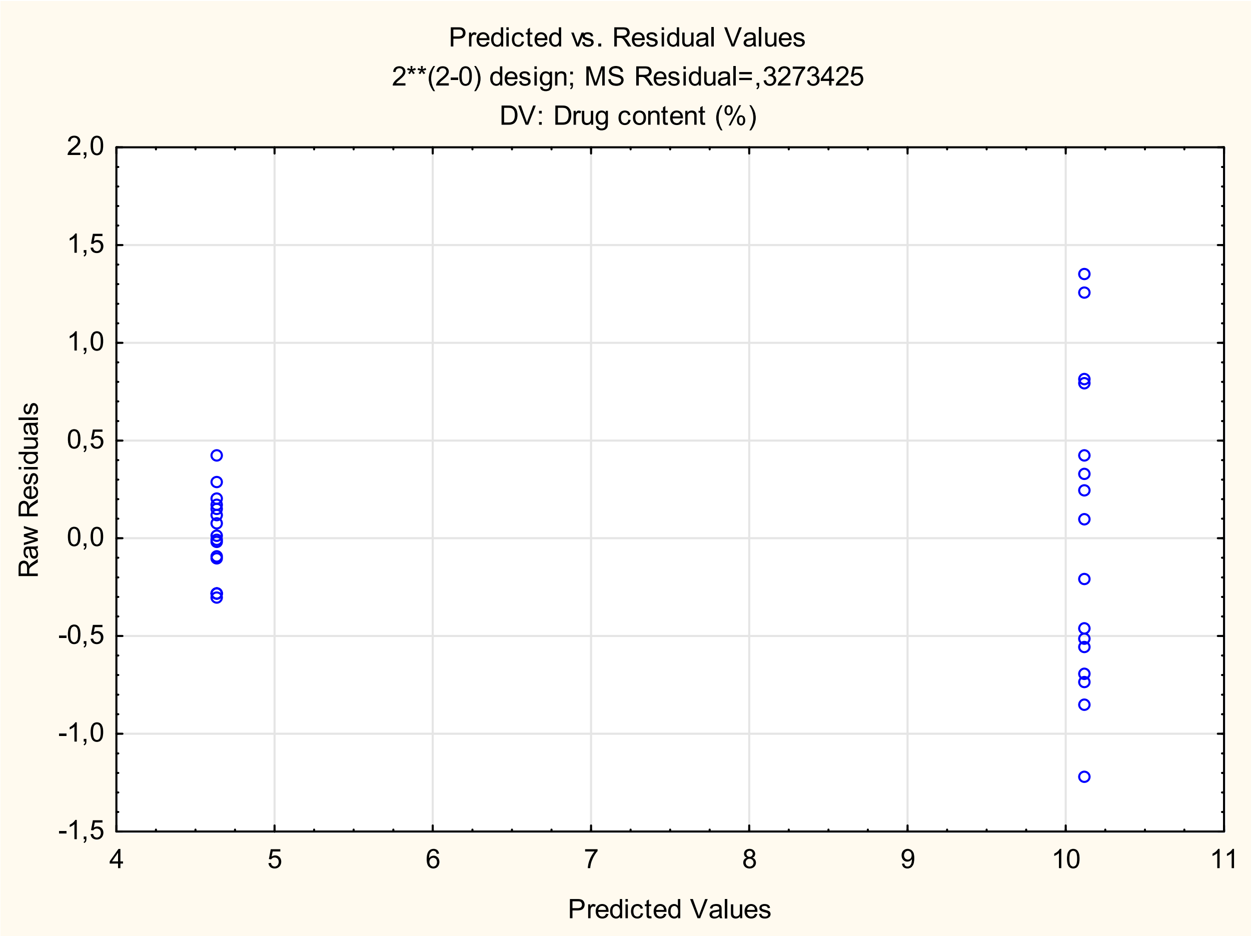

Figure S5: Residual plot analysis on drug content if all insignificant term is eliminated.

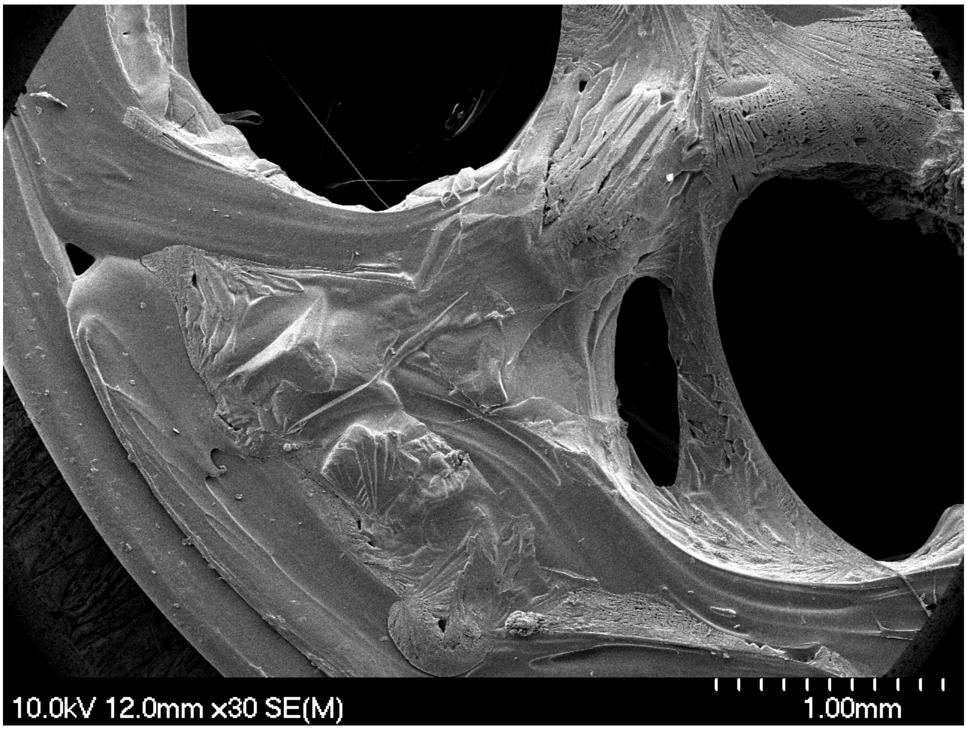

Figure S6: MS30 at 30x magnification.

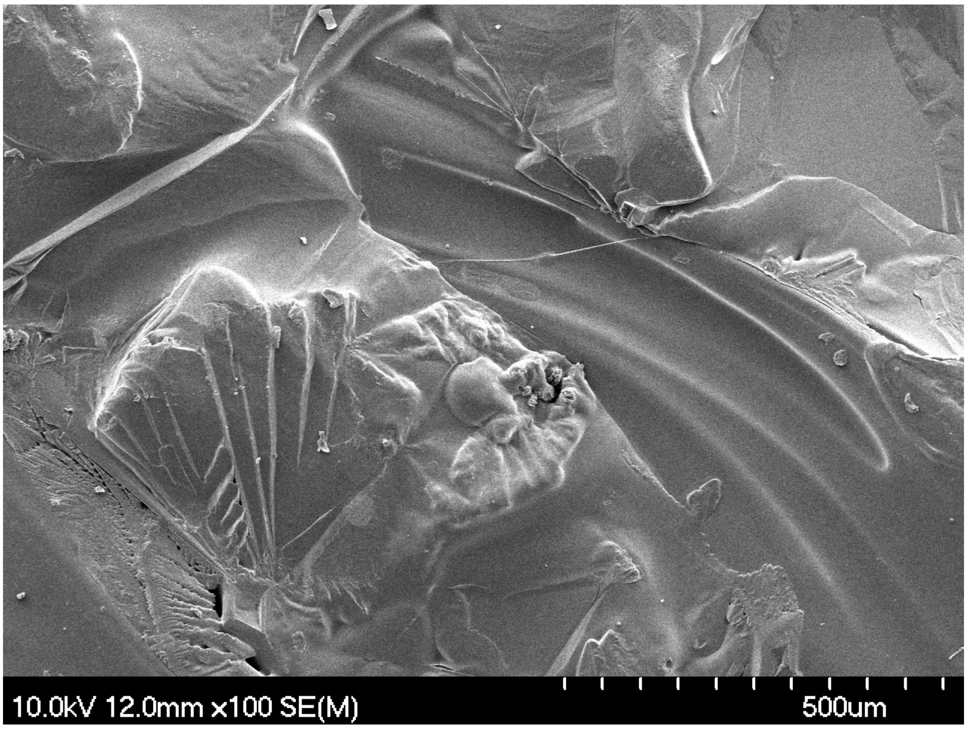

Figure S7: MS30 at 100x magnification.

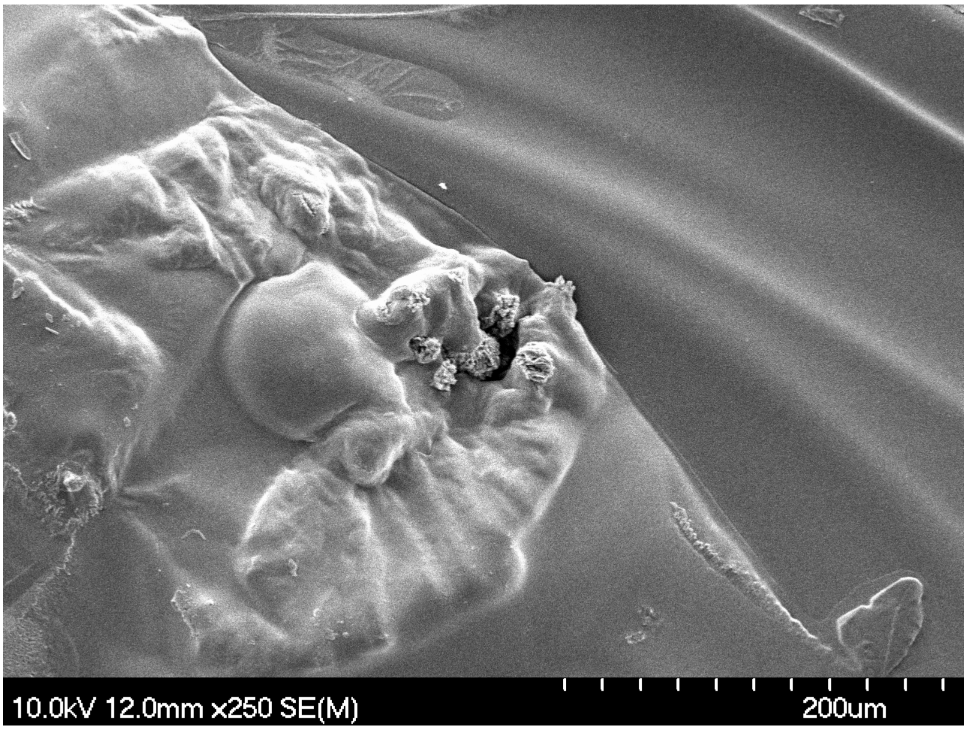

Figure S8: MS30 at 250x magnification.

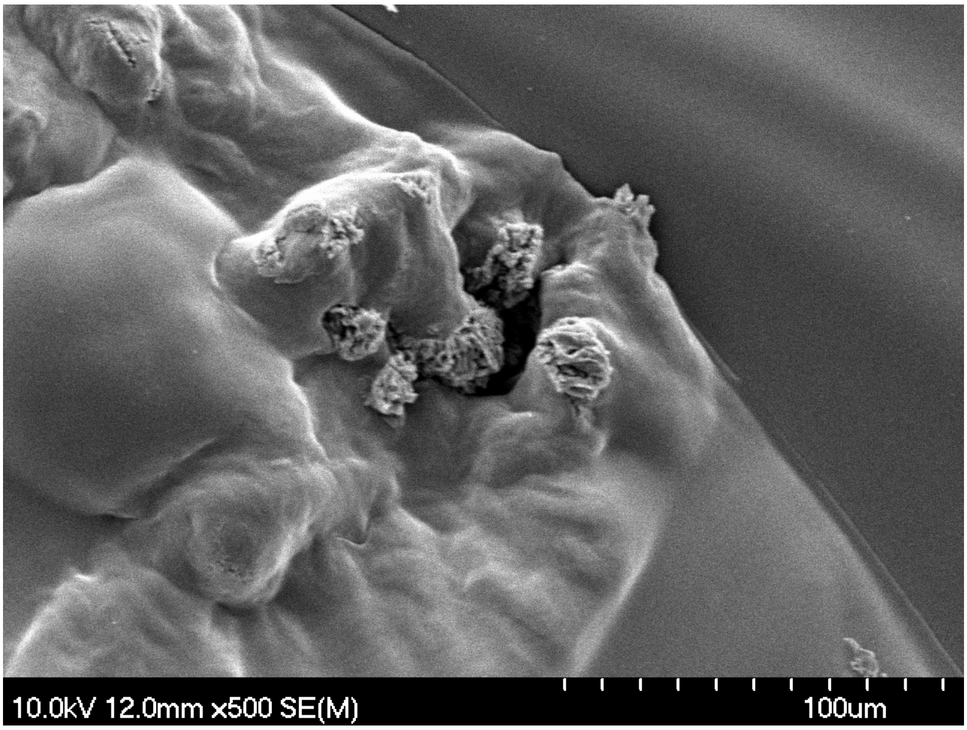

Figure S9: MS30 at 500x magnification.

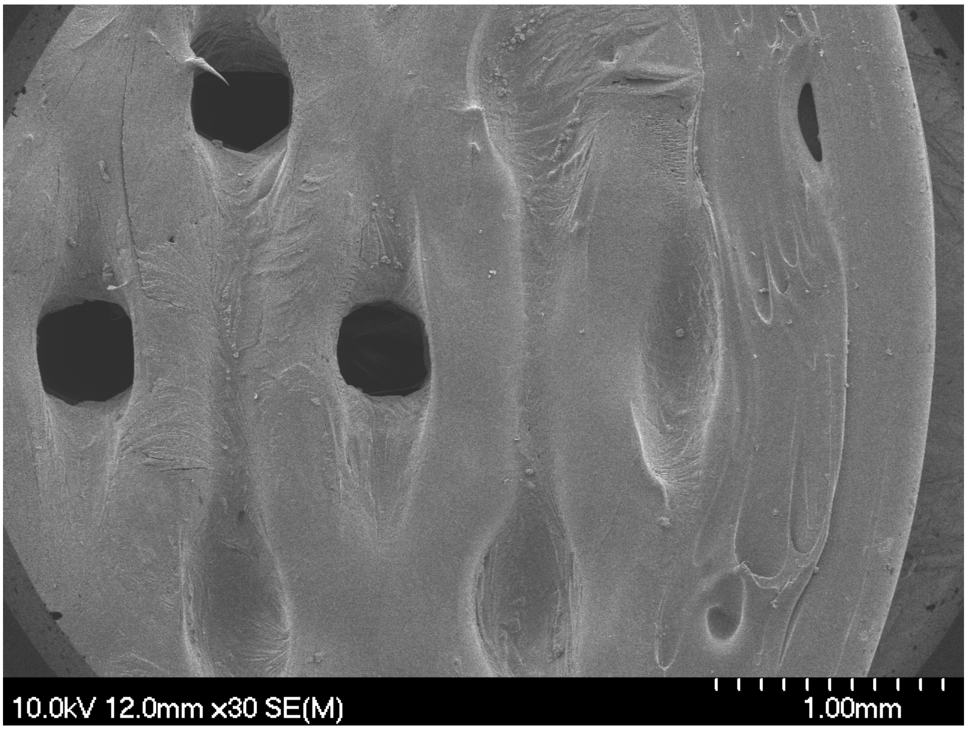

Figure S10: MS60 at 30x magnification.

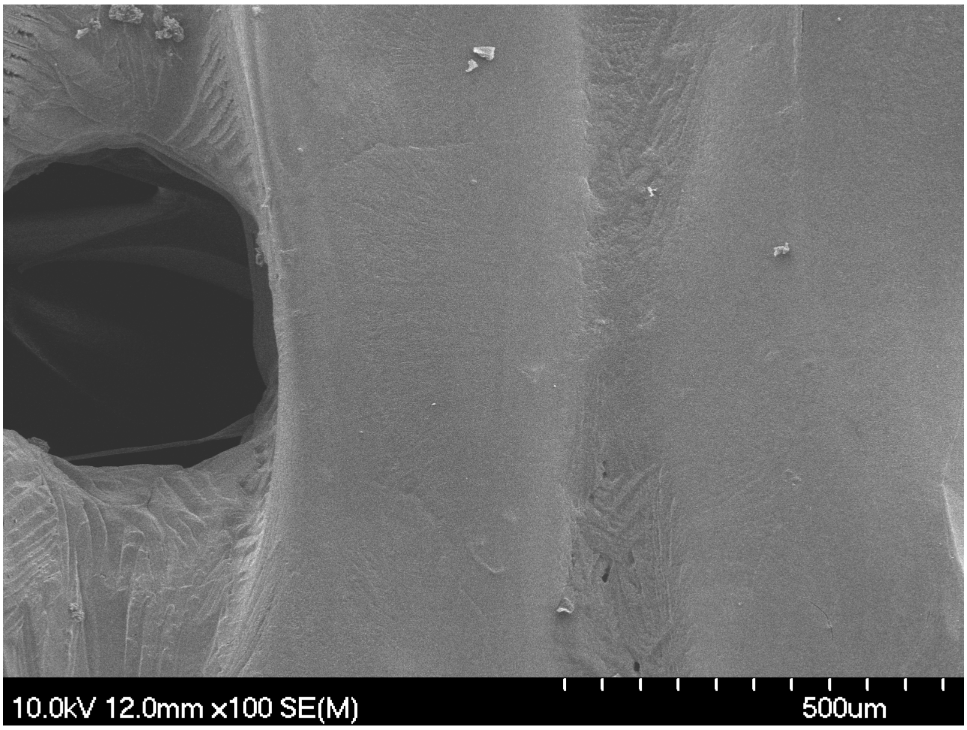

Figure S11: MS60 at 100x magnification.

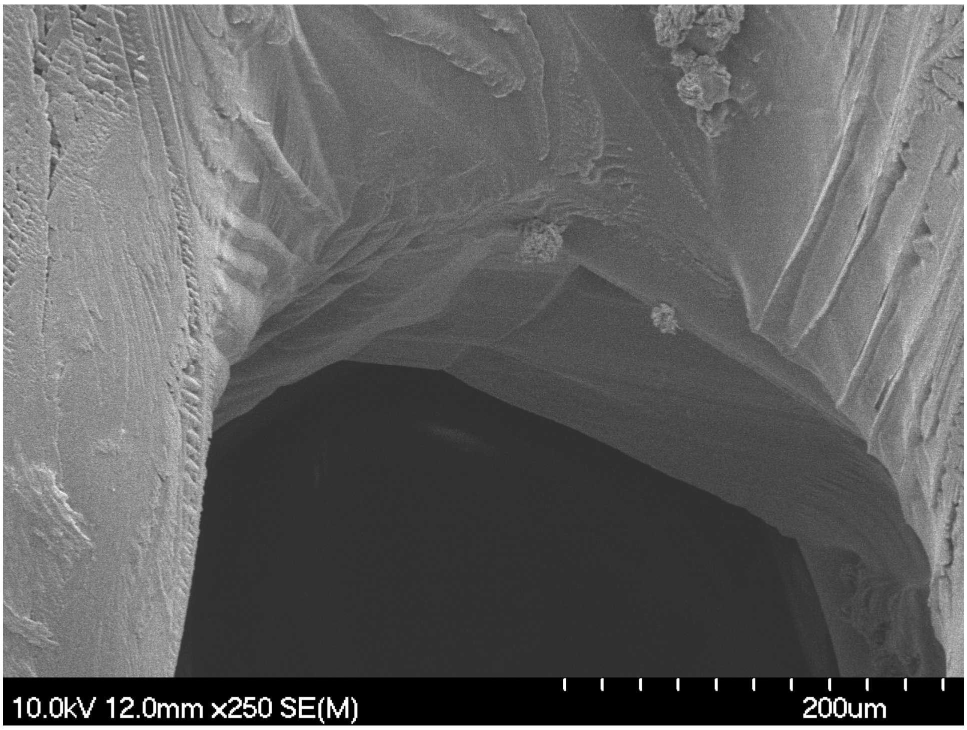

Figure S12: MS60 at 250x magnification.

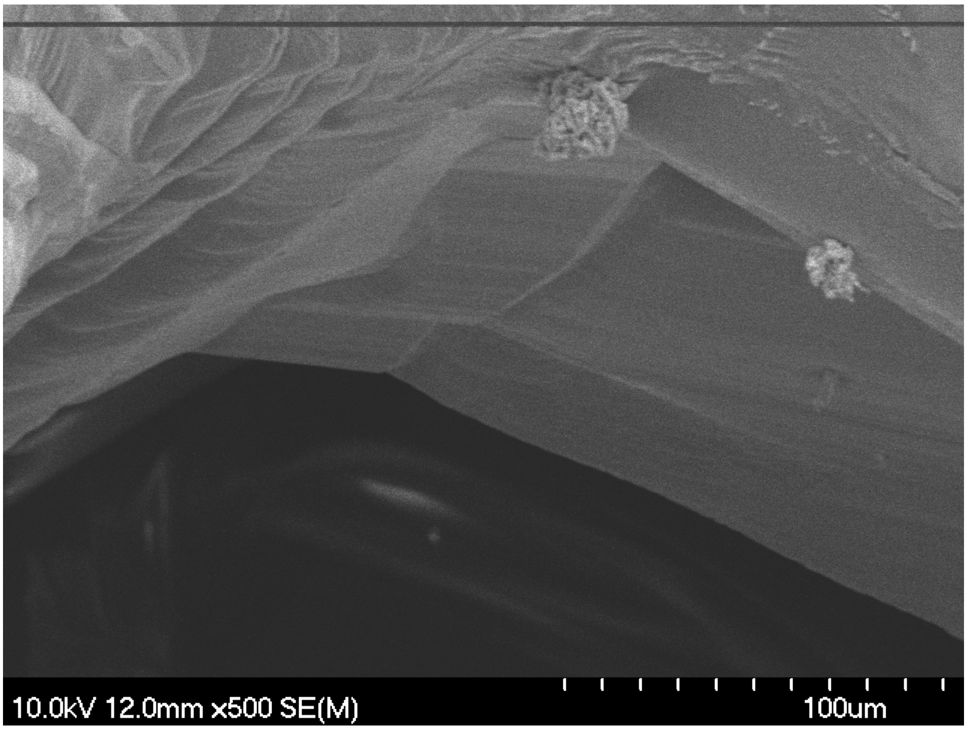

Figure S13: MS60 at 500x magnification.

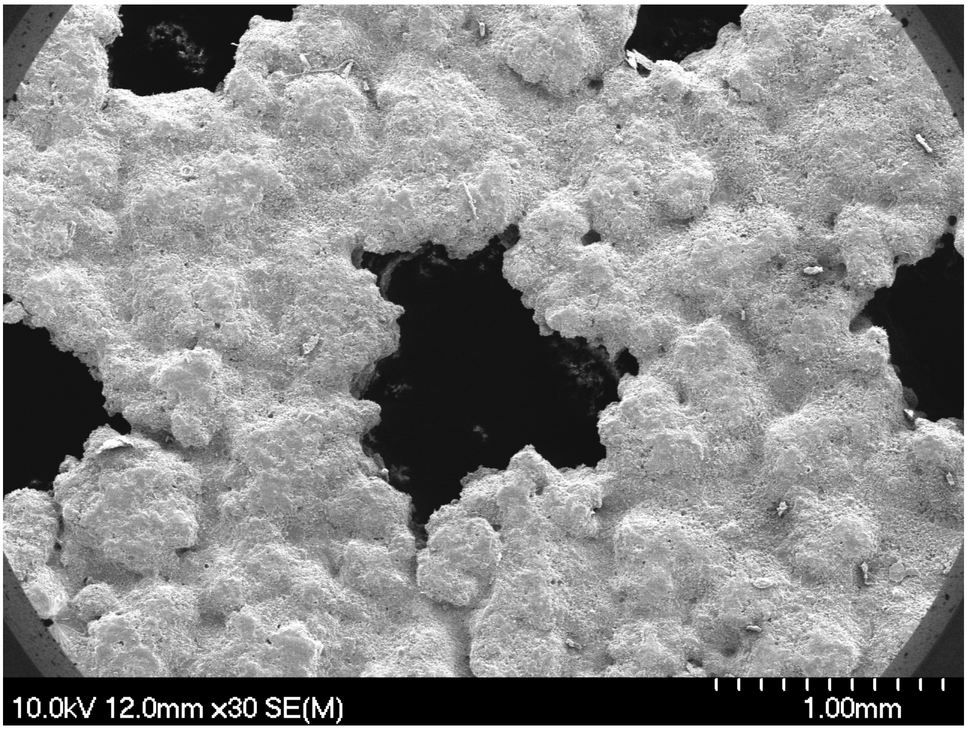

Figure S14: PC30 at 30x magnification.

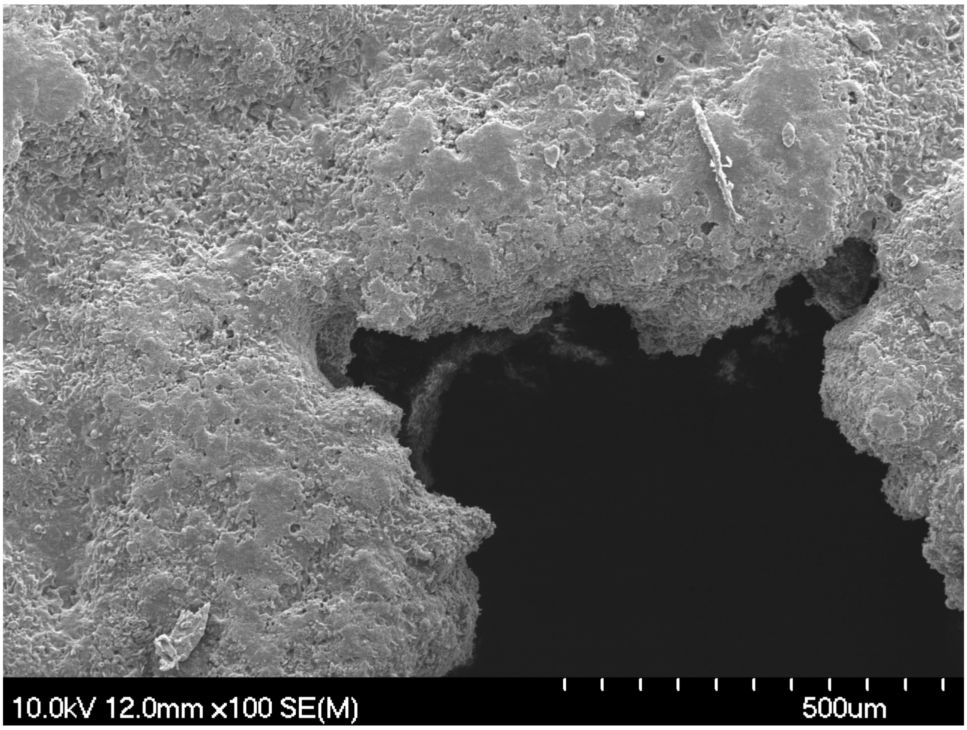

Figure S15: PC30 at 100x magnification.

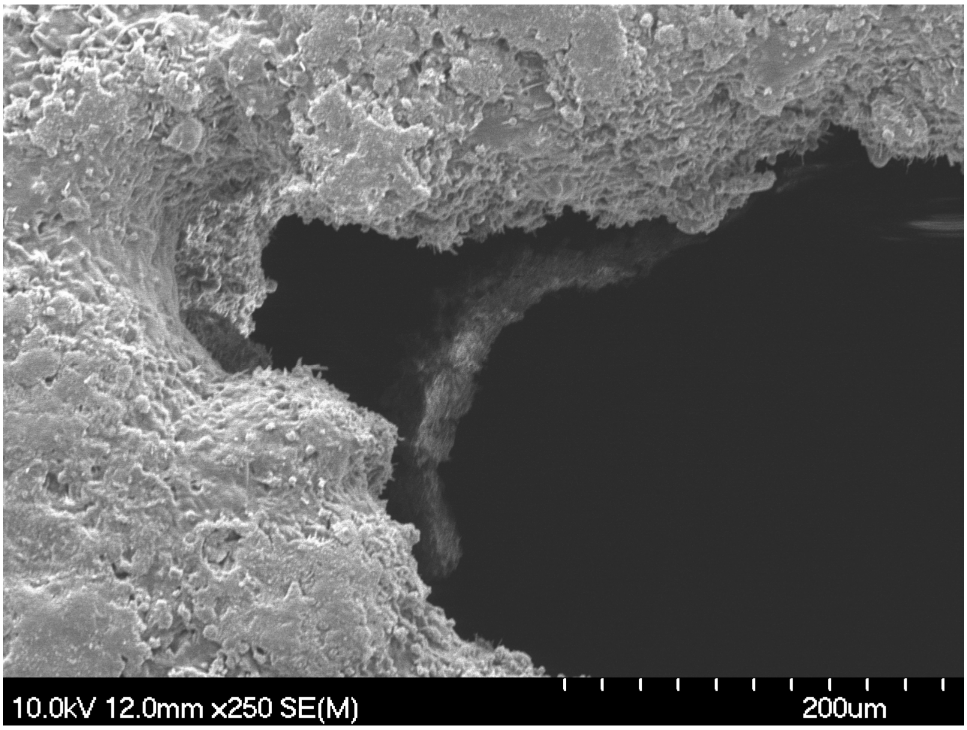

Figure S16: PC30 at 250x magnification.

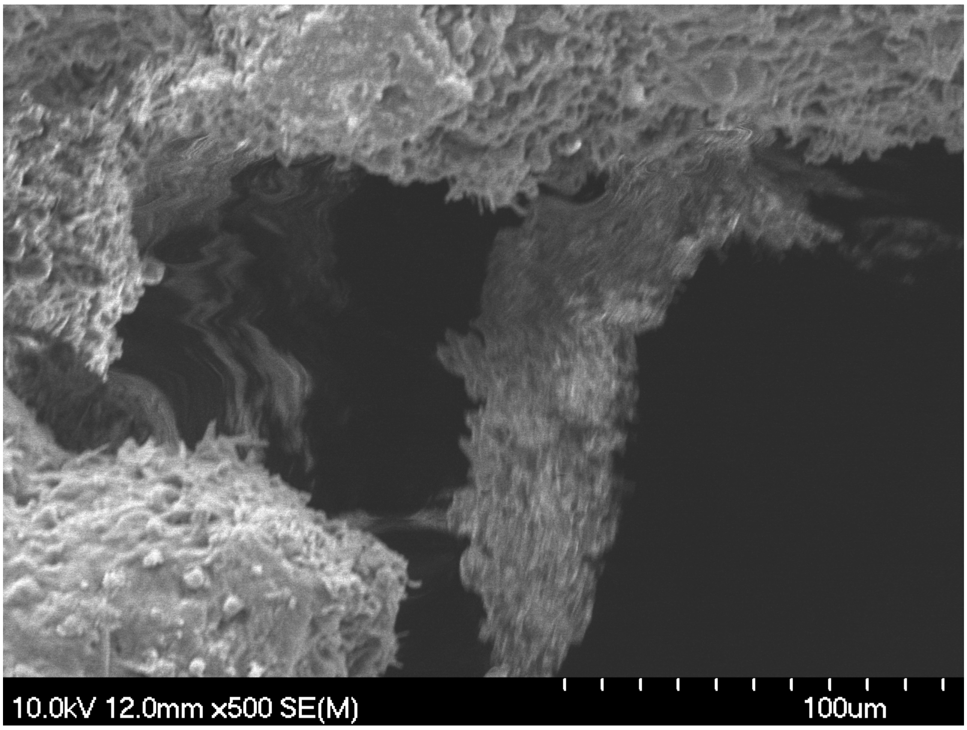

Figure S17: PC30 at 500x magnification.

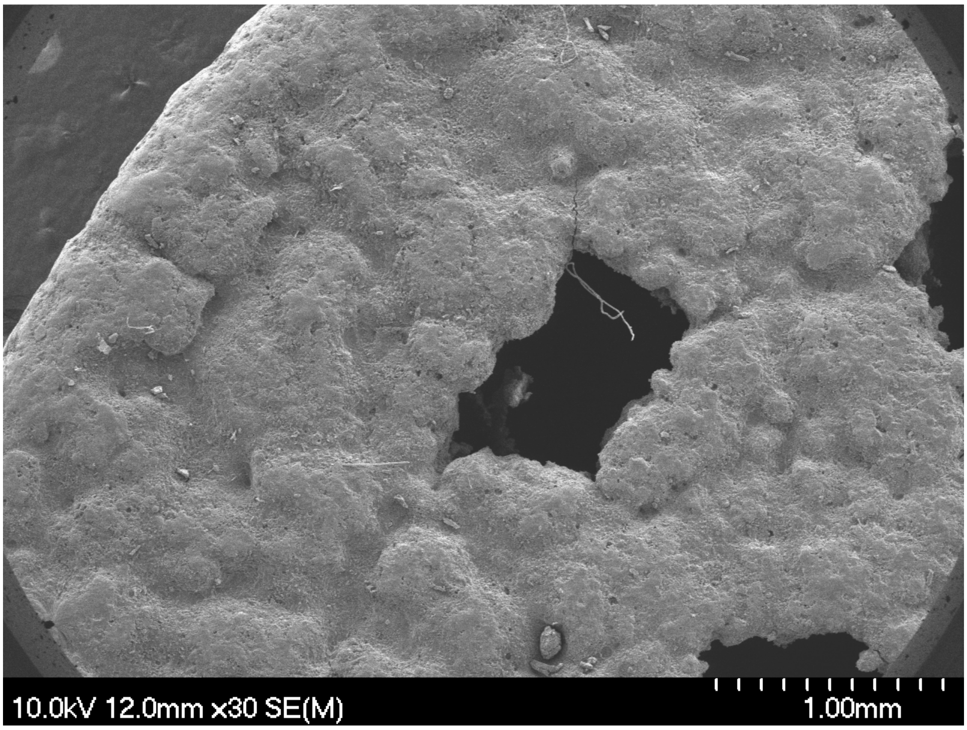

Figure S18: PC60 at 30x magnification.

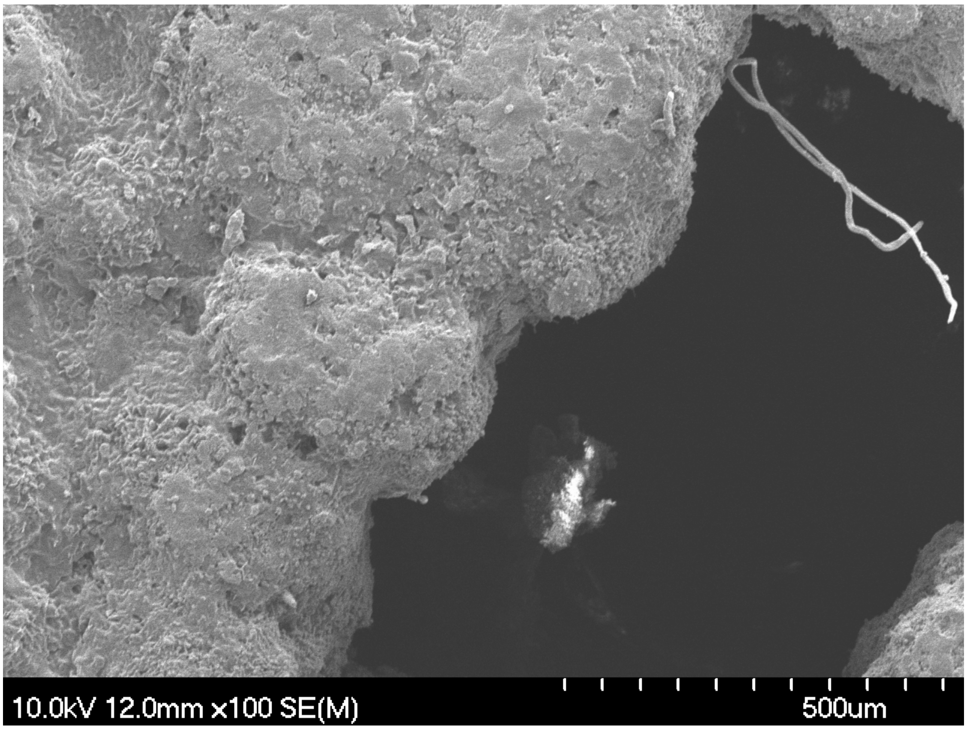

Figure S19: PC60 at 100x magnification.

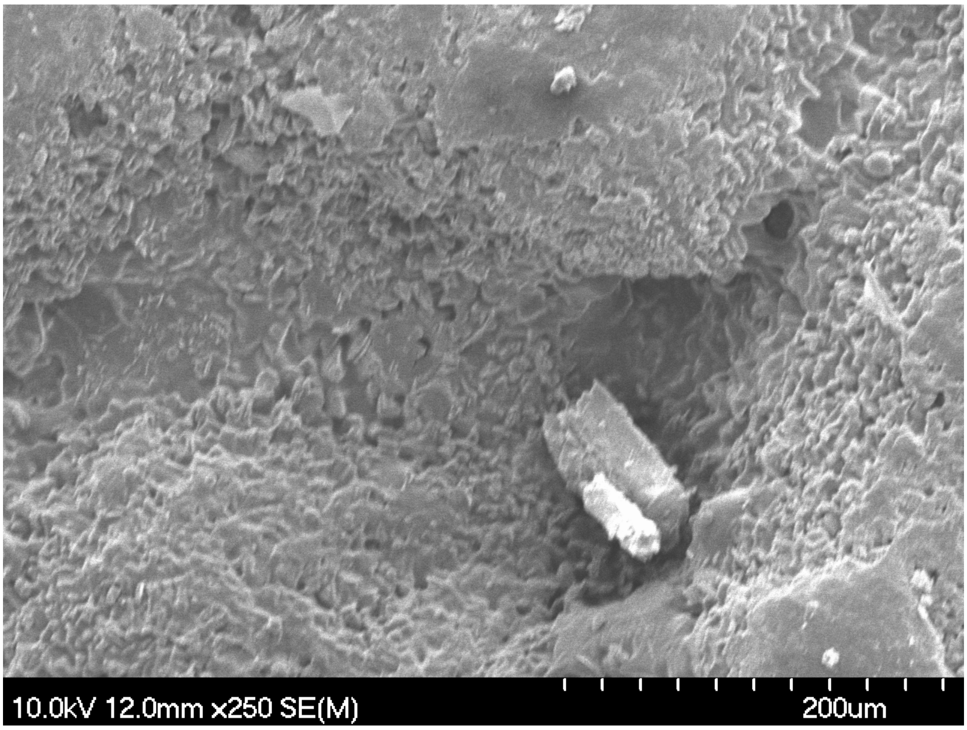

Figure S20: PC60 at 250x magnification.
